# Modeling the potential efficiency of a blood biomarker-based tool to guide pre-hospital thrombolytic therapy in stroke patients

**DOI:** 10.1007/s10198-022-01495-1

**Published:** 2022-07-27

**Authors:** Elizabeth Parody-Rua, Alejandro Bustamante, Joan Montaner, Maria Rubio-Valera, David Serrano, Soledad Pérez-Sánchez, Alba Sánchez-Viñas, César Guevara-Cuellar, Antoni Serrano-Blanco

**Affiliations:** 1 Teaching, Research and Innovation Unit, Parc Sanitari Sant Joan de Déu, Sant Boi de Llobregat, Spain; 2 Primary Care Prevention and Health Promotion Network (redIAPP), Barcelona, Spain; 3 Stroke Unit, Hospital Universitari Germans Trias i Pujol, Badalona, Spain; 4 Neurovascular Research Laboratory, Vall d’Hebron Institute of Research (VHIR), Barcelona, Spain; 5 Institute de Biomedicine of Seville, IBiS/Hospital Universitario Virgen del Rocío/CSIC/University of Seville and Department of Neurology, Hospital Universitario Virgen Macarena, Seville, Spain; 6 Head of Quality and Patient Safety, Parc Sanitari Sant Joan de Déu. Institut de Recerca Sant Joan de Déu, Sant Boi de Llobregat, Spain; 7 CIBER of Epidemiology and Public Health (CIBERESP), Madrid, Spain; 8 Freelance Health Economist, Barcelona, Spain; 9 Facultad de Ciencias de la Salud, Universidad Icesi, Cali, Colombia; 10 Parc Sanitari Sant Joan de Déu. Institut de Recerca Sant Joan de Déu, Mental Health Directorate, C/Camí Vell de la Colònia, 25, 08830 Sant Boi de Llobregat, Barcelona Spain; 11 Departament de Medicina. Facultat de Medicina i Ciències de la Salut, Universitat de Barcelona, Barcelona, Spain

**Keywords:** Stroke, Biomarkers, tPA, Cost-effectiveness

## Abstract

**Objectives:**

Stroke treatment with intravenous tissue-type plasminogen activator (tPA) is effective and efficient, but as its benefits are highly time dependent, it is essential to treat the patient promptly after symptom onset. This study evaluates the cost-effectiveness of a blood biomarker test to differentiate ischemic and hemorrhagic stroke to guide pre-hospital treatment with tPA in patients with suspected stroke, compared with standard hospital management. The standard care for patients suffering stroke consists mainly in diagnosis, treatment, hospitalization and monitoring.

**Methods:**

A Markov model was built with four health states according to the modified Rankin scale, in adult patients with suspected moderate to severe stroke (NIHSS 4-22) within 4.5 hours after symptom onset. A Spanish Health System perspective was used. The time horizon was 15 years. Quality-adjusted life-years (QALYs) and life-years gained (LYGs) were used as a measure of effectiveness. Short- and long-term direct health costs were included. Costs were expressed in Euros (2022). A discount rate of 3% was used. Probabilistic sensitivity analysis and several one-way sensitivity analyses were conducted.

**Results:**

The use of a blood-test biomarker compared with standard care was associated with more QALYs (4.87 vs. 4.77), more LYGs (7.18 vs. 7.07), and greater costs (12,807€ vs. 12,713€). The ICER was 881€/QALY. Probabilistic sensitivity analysis showed that the biomarker test was cost-effective in 82% of iterations using a threshold of 24,000€/QALY.

**Conclusions:**

The use of a blood biomarker test to guide pre-hospital thrombolysis is cost-effective compared with standard hospital care in patients with ischemic stroke.

**Supplementary Information:**

The online version contains supplementary material available at 10.1007/s10198-022-01495-1.

## Introduction

Stroke is one of the leading causes of death and disability worldwide [1, 2]. Additionally, stroke affects patients’ quality of life [3] and involves a considerable economic burden during hospitalization and subsequent discharge [3–5], mainly due to hospital stays and rehabilitation [5]. Thrombolysis with recombinant tissue plasminogen activator (tPA) for ischemic stroke (IS) is effective [6, 7] and, according to a literature review, is an efficient treatment [8]. However, it has a reduced therapeutic window (4.5 h after stroke onset) and its benefits are highly time dependent. Therefore, it is essential to confirm IS diagnosis and to treat patients promptly after symptom onset. Delays in diagnosis confirmation, which depend on neuroimaging techniques conducted on admission, are among the barriers to timely acute stroke treatment [9] and are associated with low rates of tPA treatment and poorer stroke outcomes [10]. However, symptom recognition and time to transport to hospital/accessibility to expertise would appear to greater barriers [11].

To reduce treatment delays, studies have assessed pre-hospital thrombolysis. Studies in mobile stroke units (MSUs) suggest that pre-hospital commencement of intravenous thrombolysis can improve functional outcomes in stroke patients [12–14]. MSUs are equipped with a computer tomography scanner, point-of-care laboratory, telemedicine [15, 16], stroke identification algorithm at dispatcher level, and a pre-hospital stroke team [13]. However, although MSUs have shown to be cost-effective in some studies, their great cost around 1 M€/year is unaffordable for most healthcare systems [17].

Using blood biomarkers in the ambulance to differentiate stroke types without special equipment or medical personnel would be a more practical and cheaper alternative to MSUs [14, 18]. However, the efficiency of these biomarkers is still unknown. In a recent study, our group showed the feasibility of this method by using highly specific blood tests to detect an IS patient subgroup that might benefit from pre-hospital interventions, such as intravenous thrombolysis, even without neuroimaging [19]. A two-biomarker panel including retinol-binding protein and N-terminal pro-B-type natriuretic peptide identified IS patients with 100% specificity and sensitivity rates around 20%. Moreover, a three-biomarker panel also including glial fibrillary acid protein, measured with a high-sensitivity assay, improved sensitivity to 50%, maintaining 100% specificity. These results are currently under evaluation in the BIOFAST study (Biomarkers for Initiating Onsite and Faster Ambulance Stroke Therapies, ClinicalTrials.gov identifier: NCT04612218 study), which measures these biomarkers in the pre-hospital scenario with point-of-care test devices.

This study developed a hypothetical economic model to evaluate the cost-effectiveness of these blood biomarker tests to guide pre-hospital tPA treatment vs. standard care (thrombolysis at the hospital) in patients with suspected IS.

## Methods

### Approach

A cost-effectiveness study based on a Markov model was conducted in patients with suspected stroke with < 4.5 h from symptom onset and with a National Institutes of Health Stroke Scale score of 4–22 (moderate or severe stroke). A Spanish Health System perspective was adopted. The Spanish public healthcare system provides universal coverage for citizens and foreign nationals and is mainly funded through taxes. It is a decentralized system and the 17 Spanish regions control health planning, public health, and health service management [20]. National and international economic evaluation methodological guidelines were followed [21, 22]. The 15-year time horizon was based on the mean age of stroke diagnosis (70 years) and life expectancy in Spain (85 years). A discount rate of 3% was used for both costs and outcomes [21].

### Intervention

The intervention was a biomarker test from the study by Bustamante et al. [19], consisting of the combination of retinol-binding protein > 52 µg/mL and N-terminal pro-B-type natriuretic peptide > 4062 pg/mL, providing 20% sensitivity and 100% specificity for IS (Table 1S Supplemental Material). This biomarker combination was used as base-case in the economic model. Sensitivity analyses were conducted with a three-biomarker panel including glial fibrillary acid protein, described in the same study with 51.5% sensitivity and 100% specificity for IS.

#### Comparator

The standard care for patients suffering stroke consists mainly in diagnosis, treatment, hospitalization, and monitoring. Initially, a neuroimaging test is conducted to rule out hemorrhage. In addition, analytical tests and complementary tests are done. If the stroke is ischemic and the patient meets the thrombolysis criteria, tPA IV is administered.

### Health outcomes

The main health outcome was measured in quality-adjusted life-years (QALYs) gained. This effectiveness measure was chosen because stroke patients’ functional status typically worsens considerably, which reduces health-related quality of life. Degree of disability or dependence in the daily activities of people who have suffered a stroke was measured using the modified Rankin scale (mRS) for neurologic disability. The probabilities of mRS for IS were obtained from pooled randomized controlled trials [7] that evaluated the use of tPA vs. placebo. Intracerebral hemorrhagic (ICH) mRS probabilities came from a clinical trial [23]. Regarding the biomarker test, mRS values were estimated from an MSUs study [15]. This study compared 3-month functional outcomes after intravenous thrombolysis in patients with acute ischemic stroke who had received emergency mobile care or conventional care. Previously published data on utilities were used to determine the QALY for each mRS level [24, 25].

All-cause mortality probabilities were determined by gender and age and applied to patients in the long-term phase one year after stroke, according to Spanish mortality tables, from the National Statistics Institute [26], adjusted for the relative risk of having a previous stroke. The National Statistics Institute rates were converted into probabilities. Additionally, we adjusted mortality probabilities by proportion of men (0.58) and women (0.42) suffering stroke. This information was obtained from RENISEN (National Stroke Registry of the Spanish Society of Neurology) [27, 28], a stroke registry from 42 centers in Spain. We also used LYGs as an effectiveness measure.

### Decision model

**Fig. 1:**
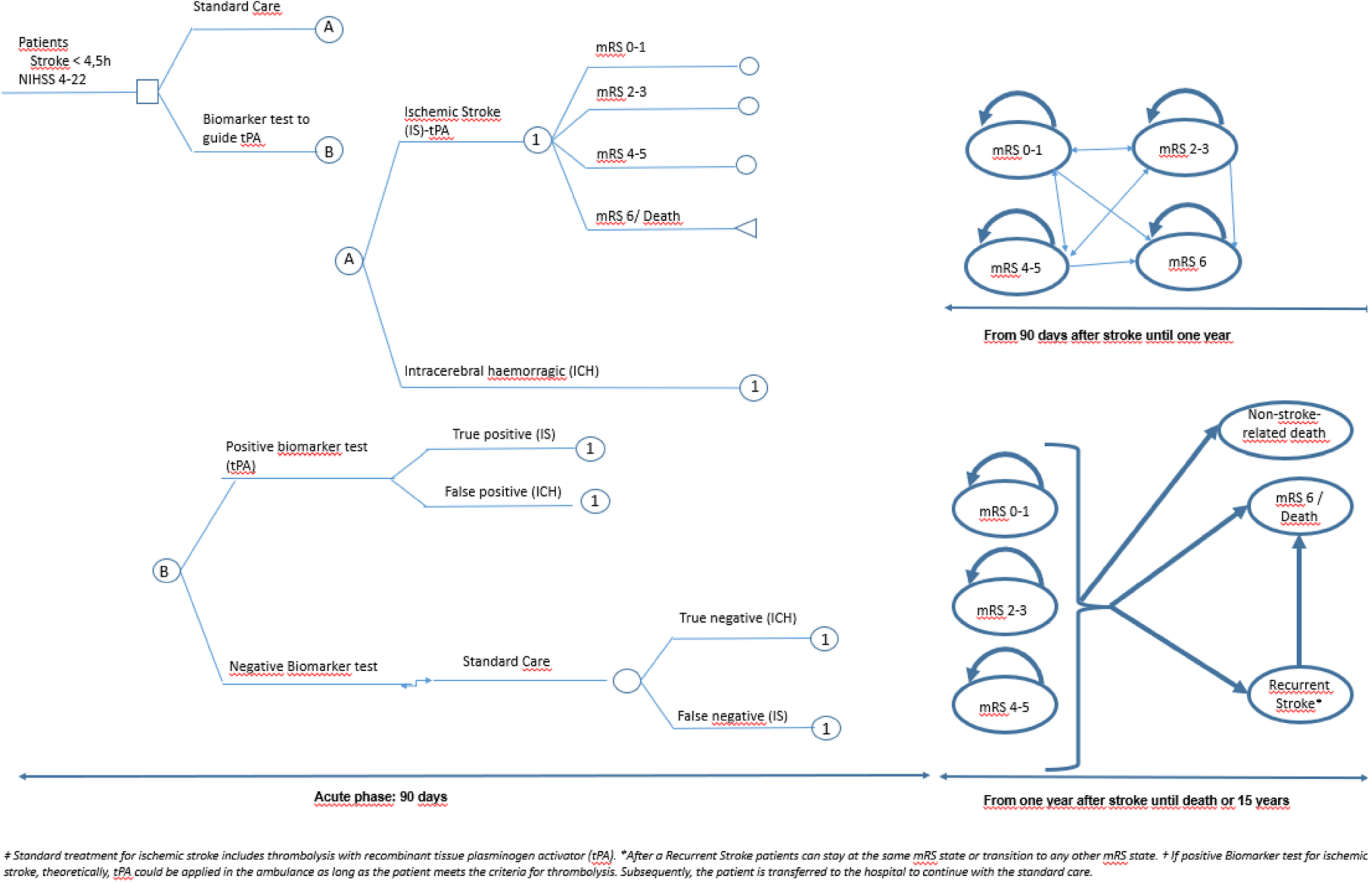
Model Structure. **A** Short-term decision analytic tree structure of clinical trial outcomes. Outcomes were: non-disabled patient, only requiring secondary prevention strategies (modified Rankin scale [mRS 0–1]), disabled patients also requiring rehabilitation (mRS 2–3), severely disabled patients, requiring long-term nursing care (mRS 4–5) and death (mRS 6) from stroke onset to 90 days. Patients enter the model with suspected stroke with < 4.5 h from symptom onset and with a National Institutes of Health Stroke Scale score between 4 and 22 (moderate or severe stroke). **B** Long-term Markov model used to simulate lifetime patient outcomes. Patients transition between the different health states as indicated by the arrows

The model was designed based on previous published model-based economic evaluations in stroke [29, 30]. We combine a decision tree with a Markov model. The model was validated by clinical experts. Markov states represent outcome categories according to mRS. Four mRS categories were used according to patients’ measurement requirements: mRS 0–1 (non-disabled patient, requiring only secondary prevention strategies), mRS 2–3 (disabled patients also requiring rehabilitation), mRS 4–5 (severely disabled patients, requiring long-term nursing care), and mRS 6 (stroke death). Figure 1 shows model structure and possible transitions between health states. The different health states were defined to be clinically and economically relevant. The model has a two-phase structure: acute (short run represented by a decision tree model) and long term (Markov model). The acute phase consists of patient management and outcomes from stroke onset to 90 days. The model then follows a long-term phase with two different cycle lengths: 91 days to a year after stroke and from one year to 15 years with an annual cycle length; therefore, a half-cycle correction was made.

The model was tested for construct validation, verification, and cross-validation by clinical experts (JM, AB) and evaluated using a first-order Monte Carlo simulation in Microsoft Excel 2016.

Model assumptions were as follows: (1) the proportion of patients undergoing standard care vs. test biomarker was 50%; (2) when the test biomarker is positive for IS, theoretically, tPA could be applied in the ambulance if the patient meets the criteria for thrombolysis. Subsequently, the patient would be transferred to the hospital to continue with standard care; (3) probabilities of health-state transition were the same for both groups; (4) after the first year of stroke, patients remained in the same mRS unless they had a recurrent stroke or died; (5) in case of recurrence, the same type of stroke was assigned; and (6) a conservative risk option of annual recurrence was adopted and we assumed that recurrent stroke rates were equal across all categories of mRS, in accordance with previous studies [24, 25].

Table 1 shows the model parameters and distributions.

**Table 1:** Model input parameters with base-case values and distribution probabilities used in the sensitivity analysis

Parameter	Base-case value	Type of distribution	Parameter 1 of distribution^a^		Parameter 2 of distribution^b^		Source(s)	
Standard care probabilities IS at 3 months								
mRS 0–1	0.42	β	194		269		[7]	
mRS 2–3	0.21	β	97		366			
mRS 4–5	0.19	β	88		375			
mRS 6	0.18	β	83		380			
Biomarker test alternative probabilities IS at 3 months								
mRS 0–1	0.50	β	220		218		[15]	
mRS 2–3	0.25	β	110		328			
mRS 4–5	0.14	β	61		377			
mRS 6	0.11	β	48		390			
Both alternatives probabilities ICH at 3 months								
mRS 0–1	0.10	β	9		84		[23]	
mRS 2–3	0.34	β	32		61			
mRS 4–5	0.33	β	31		62			
mRS 6	0.23	β	21		72			
Annual probability of recurrent stroke	0.05	β	5		95		[32]	
Probabilities of transition in the first year								
Probability from mRS 0–1 to:								
mRS 0–1	0.70	β	66		28		[33]	
mRS 2–3	0.07	β	7		87			
mRS 4–5	0.09	β	8		86			
mRS 6	0.14	β	13		81			
Probability from mRS 2–3 to:								
mRS 2–3	0.33	β	24		48		[33]	
mRS 4–5	0.13	β	9		63			
mRS 6	0.14	β	10		62			
mRS 0–1	0.40	β	29		43			
Probability from mRS 4–5 to:								
mRS 4–5	0.55	β	31		25		[33]	
mRS 6	0.30	β	17		39			
mRS 0–1	0.02	β	1		55			
mRS 2–3	0.13	β	7		49			
Utilities								
mRS 0–1	0.84	β	85		15		[25]	
mRS 2–3	0.72^c^	β	73		27		[24]	
mRS 4–5	0.45^c^	β	41		58		[24]	
Costs^d^								
mRS 0–1 IS	8,101. 51	γ	81,015.10		0.1		Regional Government Official Bulletins; Catalonia hospital; Andalucia hospital; BotPlus 2019; [34]; [35]	
mRS 2–3 IS	11,099.88	γ	110,991.80		0.1			
mRS 4–5 IS	16,257.44	γ	162,574.40		0.1			
mRS 6 IS^e^	6,787.84	γ	67,878.40		0.1			
mRS 0–1 ICH	7,992.68	γ	79,926.80		0.1		Regional Government Official Bulletins, Catalonia hospital; Andalucia hospital; BotPlus 2019; [35]; [36]	
mRS 2–3 ICH	14,048.47	γ	140,484.70		0.1			
mRS 4–5 ICH	16,758.86	γ	167,588.60		0.1			
mRS 6 ICH^e^	10,172.30	γ	101,723.00		0.1			
Biomarker blood test	101.04	γ	1,010.40		0.1		BIOFAST	

*IS* ischemic stroke, *ICH* intracerebral hemorrhagic

^a^Parameter 1 is: alpha (beta and gamma distribution), lower value (uniform distribution)

^b^Parameter 2 is: beta (beta and gamma distribution), upper value (uniform distribution)

^c^Utility by time trade-off (TTO) method

^d^Euros 2022

^e^Only hospitalization costs

### Use of resources and costs

In line with the study perspective, direct health costs were included using a micro-costing strategy. Five aspects were considered for cost estimations: (1) Comparison of the alternatives. The biomarker test was the differentiating cost. All other resources were considered to be the same, except for the proportion of resources used; (2) Type: IS or ICH; (3) Functional status according to mRS; (4) Cost-allocation time: acute phase, discharge after three months, three months during the first year and first year, long-term; and (5) Destination at discharge, to allocate corresponding costs for institutionalized patients.

Clinical and economic evaluation studies on stroke were used as a basis for resource use according to the five aforementioned aspects. An electronic survey was used to determine the use of clinical resources in different Spanish hospitals. Twenty-eight neurologists from eight regions participated, of whom 13 answered all the questions. Finally, four stroke neurologists validated the information on resource use.

Acute phase costs were as follows: neuroimaging, laboratory tests, length-of-stay including inpatient care, pharmacological treatment (tPA and other drugs according to stroke type), and rehabilitation (physiotherapy, speech therapy and occupational therapy, the proportion of patients, and the number of sessions). We included the cost of treatment for symptomatic hemorrhagic transformation as an adverse event from tPA administration (2% of patients, according to data from Catalonia’s Database-CICAT).

Costs included from discharge to 90 days and from 90 days to one year were: follow-up outpatient visits with the neurologist and/or primary care physician, rehabilitation, additional orthopedic resources covered by the health system (wheelchair, walker, or cane), follow-up neuroimaging, and laboratory tests. For costs from one year onward, we considered the cost of stroke recurrence and follow-up outpatient visits where necessary, according to survey results and validation by stroke physicians/clinical experts. It was assumed that patients are prescribed an annual blood test.

Length-of-stay costs were included for patients whose destination at discharge was a convalescent facility or long-term stay. Patients in a long-term care facility one year after the stroke were assumed to remain there for the rest of their lives.

Total cost was calculated by multiplying the proportion of resources used by their unit cost and by the units required of each resource. Public health service tariffs are published in Regional Government Official Bulletins, updated to 2022 using the specific regional healthcare consumer price index. Drug costs were obtained from BootPlus 2022. Costs were expressed in euros (2022). Table 2 shows the costs per health state.

**Table 2:** Costs of treatment (in 2022 euros) per stroke subtype and modified Rankin scale (mRS)

mRS/resource	Ischemic stroke	Intracerebral hemorrhagic
mRS 0–1		
Acute phase	6651.39	6410.48
Discharge to 90 days	759.88	821.94
90 days to one year	495.78	558.78
After one year	194.46	201.48
Stroke recurrence	4500.60	5739.49
mRS 2–3		
Acute phase	8019.64	10,438.20
Discharge to 90 days	2139.85	2637.23
90 days to one year	694.88	715.20
After one year	244.81	257.84
Stroke recurrence	6053.03	9824.67
mRS 4–5		
Acute phase	10,067.72	10,589.38
Discharge to 90 days	4433.52	4624.48
90 days to one year	941.03	1090.78
After one year	444.69	454.22
Stroke recurrence	8168.39	9925.05

### Deterministic and probabilistic sensitivity analysis

We conducted an analysis to determine the incremental cost-effectiveness ratio (ICER).

ICER is expressed mathematically in the following expression:

$$ICER=\frac{Incremental cost}{Incremental QALYs}=\frac{{Cost}_{B}-{Cost}_{A}}{{QALY}_{B}-{QALY}_{A}}.$$

In this study, B is the intervention and *A* the comparator.

Deterministic one-way sensitivity analyses were performed to identify variables significantly influencing model outcomes: (1) test sensitivity and specificity, (2) using a three-biomarker panel which includes glial fibrillary acid protein, (3) test cost, and (4) changing thrombolysis treatment (tPA for tenecteplase—TNK). This entailed modifications in mRS probabilities and costs in the economic model. TNK is non-inferior to tPA as a treatment for stroke patients [35] with similar safety profiles [38]. We used mRS probabilities with TNK from a meta-analysis [37] compared with standard care. For the biomarker test strategy, we estimated the probabilities from Kunz et al. [15].

A probabilistic sensitivity analysis (PAS) with second-order Monte Carlo simulation with 1000 iterations was performed. A willingness-to-pay threshold of 24,000€/QALY was considered consistent with national recommendations [39]. The efficiency threshold in the PAS has been changed, using values of 22, 25, 30, and 60 thousand €/QALY [39, 40].

## Results

Table 3 shows the deterministic cost-effectiveness analysis and one-way sensitivity analysis. The blood test biomarker used to guide pre-hospital tPA administration compared with standard care was associated with more QALYs (4.87 vs. 4.77), more LYGs (7.18 vs. 7.07), and greater costs (12,807€ vs. 12,713€). The ICER was 881€/QALY in the base-case scenario.

**Table 3:** Cost-effectiveness analyses of the biomarker test to guide pre-hospital tPA: base-case scenario and sensitivity analyses

Alternatives	Costs (€)	Incremental Cost	QALY	Incremental QALY	ICER (€/QALY)	LYG	Incremental LYG	ICER (€/LYG)
Base-case scenario (S = 20%)^a^								
Standard care	12,713		4.77			7.07		
Biomarker test	12,807	94	4.87	0.11	881	7.18	0.11	822
Test sensitivity = 30% ^a^								
Standard care	12,713		4.77			7.07		
Biomarker test	12,818	105	4.92	0.16	662	7.24	0.17	618
Test Sensitivity = 10% ^a^ (hypothetical worst scenery)								
Standard care	12,713		4.77			7.07		
Biomarker test	12,794	81	4.83	0.06	1369	7.13	0.06	1286
Test sensitivity = 60% ^a^ (hypothetical best scenery)								
Standard care	12,713		4.77			7.07		
Biomarker test	12,862	139	5.09	0.32	433	7.41	0.34	404
Test sensitivity = 56.5% and Specificity = 68%^b^								
Standard care	12,713		4.77			7.07		
Biomarker test	14,692	1979	4.83	0.06	33,045	6.99	-0.07	Dominated
Using three biomarkers (Sensitivity = 52%; Specificity = 100%)								
Standard care	13,611		4.45			6.86		
Biomarker test	13,742	130	4.61	0.16	820	7.04	0.17	760
Doubling the cost of the test (200€)^a^								
Standard care	12,713		4.77			7.07		
Biomarker test	12,893	180	4.87	0.11	1576	7.18	0.11	1689
Changing alteplase for tenecteplase (TNK)^a^								
Standard care	13,089		5.66			7.99		
Biomarker test	13,156	67	5.73	0.07	956	8.05	0.07	1027

*S* sensitivity, *E* specificity, *QALY* quality adjusted-life year, *ICER* incremental cost-effectiveness ratio, *LYG* life-year gained

^a^Prevalence of ischemic stroke was 81.9% and of hemorrhagic stroke was 18.1%. Specificity = 100%

^b^Prevalence of ischemic stroke was 49.3% and of hemorrhagic stroke was 50.7%

The univariate sensitivity analysis revealed that when test sensitivity increased (e.g., to 30%), QALY and LYG also increased. The test was cost-effective in all scenarios, except when test sensitivity was 56.5% and test specificity was 68%. In this case, the biomarker test had an ICER of 33,045€/QALYs and was a dominated strategy (more costly and fewer LYGs compared with standard care). Using three biomarkers (52% sensitivity and 100% specificity), the ICER was 820€/QALYs.

**Fig. 2:**
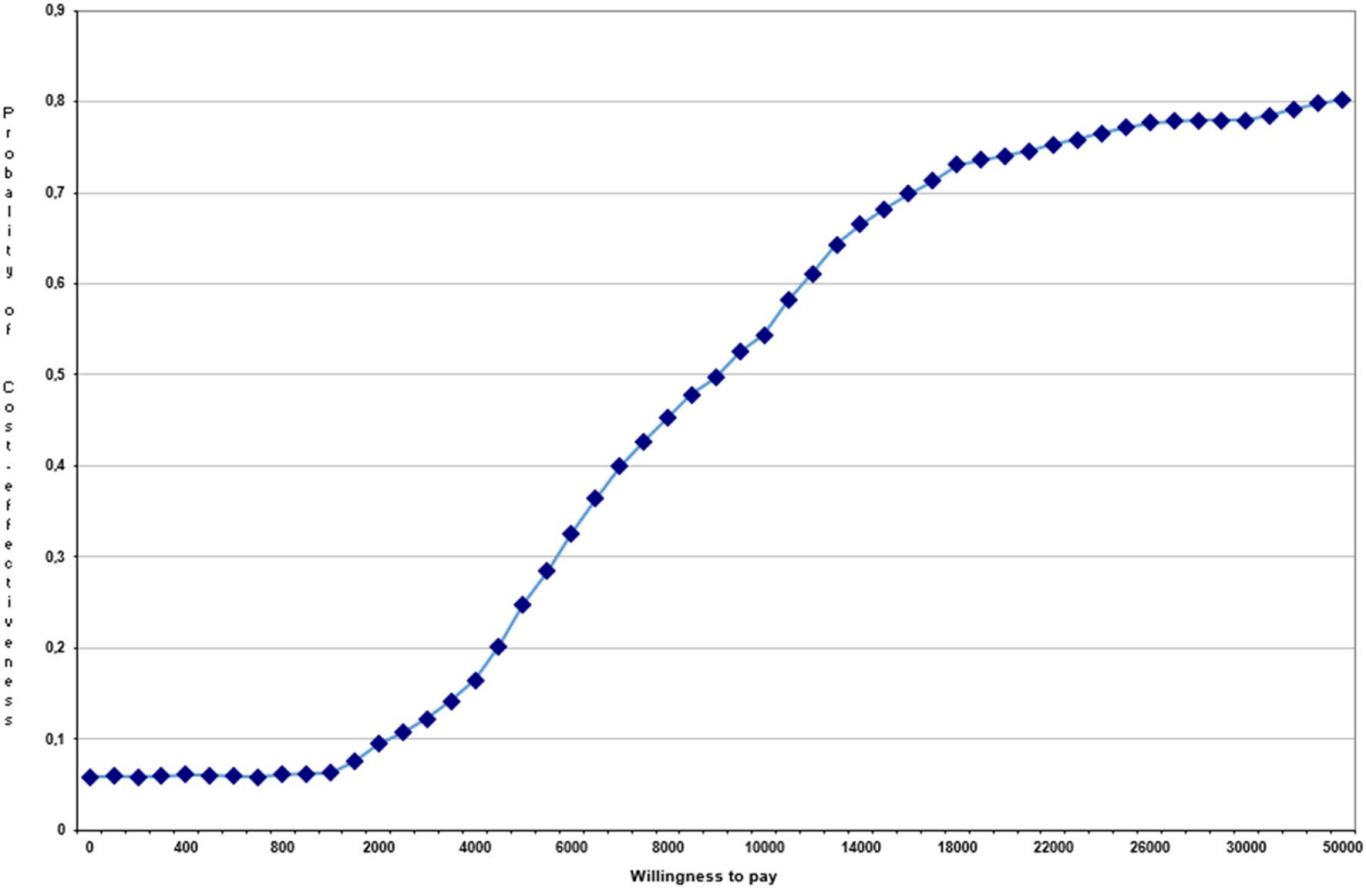
Cost-effectiveness acceptability curve

Figure 2 represents the cost-effectiveness acceptability curve. The test biomarker presented a greater probability of being cost-effective for willingness-to-pay thresholds higher than 10,000€/QALY. For a willingness-to-pay of 24,000€/QALY, the test biomarker presented an 82.5% probability of being cost-effective.

We ran several models applying different blood test sensitivity and specificity. The higher the sensitivity of the test, the lower the ICER (Fig. 1S Supplemental Material) and QALYs ranged from 0.06 to 0.52 with test sensitivities of 10% and 99%, respectively (Fig. 2S Supplemental Material). When the cost of the test was changed using different sensitivity and specificity values (according to Bustamante’s study [19] and hypothetical values), the test was cost-effective when its unit price was 10,000€ and had a sensitivity of 99% and specificity of 100%. As the sensitivity decreases, the ICER is no longer cost-effective despite lowering the cost of the test; for instance, a test with a sensitivity of 10% that costs 2000€ would no longer be effective (Table 4). Table 2S (Supplemental Material) shows the maximum individual cost of the test to make it a cost-effective alternative; it is observed that this cost varies from 874 to 13,986€, values that depend on the diagnostic validity used.

**Table 4:**
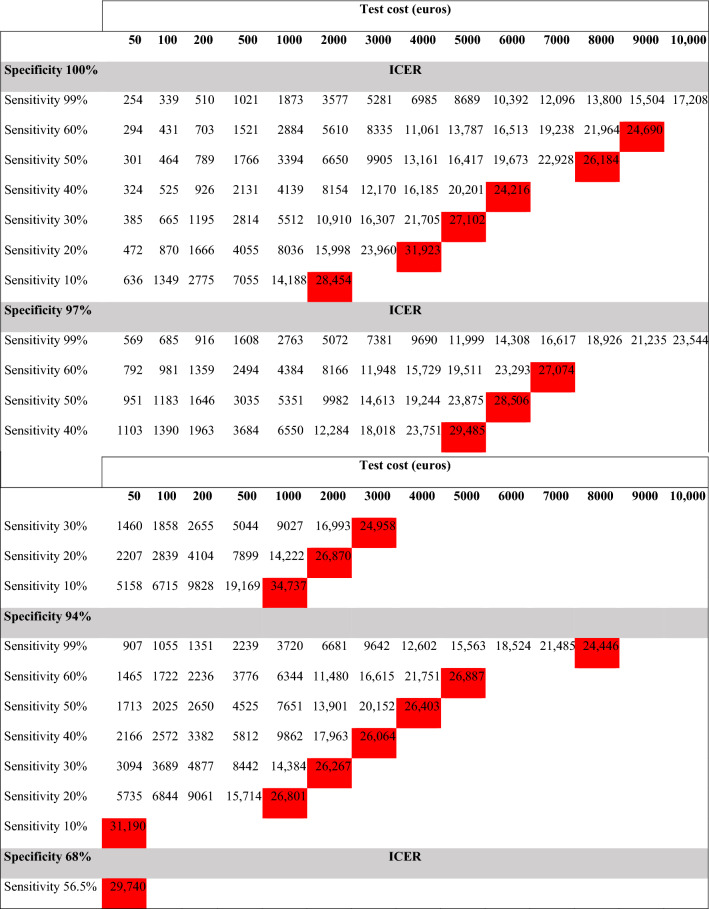
Incremental cost-effectiveness ratio (ICER) according to cost, sensitivity, and specificity of the biomarker test

Values in red are not cost-effective

ICER: incremental cost-effectiveness ratio

The incremental cost-effectiveness plane is provided in Supplementary Materials (Fig. 3S). The Monte Carlo simulation shows that most of the simulations are within the confidence ellipse, showing that the results are quite robust to changes in the values of the different variables simultaneously.

The biomarker test was cost-effective in 82.4%, 82.5%, 82.8%, and 83.1% of interactions when the willingness-to-pay threshold was changed by 22,000, 25,000, 30,000, and 60,000 €/QALY, respectively.

## Discussion

Our study shows that using a pre-hospital blood test to guide tPA may be a cost-effective strategy in a hypothetical cohort of patients with suspected moderate to severe stroke (NIHSS 4-22) within 4.5 h after symptom onset from the Spanish Health System perspective.

This is among the first economic evaluations of biomarker tests for guiding tPA in the ambulance in patients with IS. However, previous studies showed the efficiency of pre-hospital treatment for acute stroke. An economic model study found that the MSUs strategy could be cost-effective in different scenarios, although efficiency was related to population density [41]. The MSUs strategy led to a higher frequency of thrombolysis and a higher proportion of patients in the early time interval (within 90 min: 48.1% vs. 37.4%; 91–180 min: 37.4% vs. 50%; 181–270 min: 14.5% vs. 12.8%), which resulted in 32,456€/QALY [42].

In the MSUs strategy, the ambulance was equipped with a scanner, a point-of-care laboratory, and telemedicine capabilities, making the cost prohibitive for routine clinical implementation in many countries [42]. In our economic model, the ambulance cost is the same as in usual care, and the biomarker cost (around 100€/unit) is easily affordable. Administration of tPA in ambulances is a usual practice in the drip-and-ship model [43], with the exception that tPA is initiated at primary stroke centers, while the 1-h intravenous infusion is continued at the ambulance. Therefore, paramedics are familiar with tPA infusion and able to recognize tPA-related complications, such as bleedings or angioedema. However, tPA initiation at conventional ambulances might require an extra inversion in educational aspects. This education would also improve one of the greater barriers to early treatments, which is stroke symptoms’ recognition. In the sensitivity analysis, when the test unit price was doubled, it was still an efficient alternative.

In the base-case and all one-way sensitivity analyses in this study, QALYs were higher for the biomarker strategy vs. standard care, mainly due to shortening of tPA administration time, resulting in more patients with a favorable mRS [16]. Accordingly, a study showed that in acute IS patients who were treated with tPA, shorter door-to-needle times (within 45 min) were associated with lower all-cause mortality and lower all-cause readmission at 1 year [44].

The economic model used preliminary sensitivity (20%) and specificity (100%) results from the biomarker study [20]. When the sensitivity is low, patients who are not diagnosed in the ambulance are not compromised, as they follow the same current stroke pathways and would be transferred to a hospital where stroke diagnosis is made by neuroimaging. The test (which takes about 15 min) needs no additional time as it is performed during the transfer. On the other hand, when specificity values were decreased, the number of false positives increases; in these cases, patients with IH are treated with tPA with the risk of worsening. Lower specificity values were included in the model in sensitivity analysis. It is important to note that in addition to clinical enforcement and economic impact, ethical dilemmas would arise. Regarding the sensitivity analysis with the three-biomarker panel, results should be carefully interpreted, as this substudy was planned as a 1:1 case–control study (one ischemic vs. one hemorrhagic patient). In our economic model, test accuracy depends on stroke subtype prevalence. When we used the three-biomarker panel, the prevalence was 49.3% and 50.7% of ischemic and ICH, respectively. Here, the test was cost-effective but with fewer QALYs gained (0.16) compared with the same sensitivity and specificity using two biomarkers (0.27 QALYs).

The use of TNK in patients with stroke in the economic model showed more QALY and LYG gained in both alternatives, and the test was cost-effective using this thrombolytic. Furthermore, the use of TNK has the advantage that it is easier to administer (in bolus) than tPA perfusion in the ambulance.

### Strengths and limitations

This is the first economic evaluation of pre-hospital blood test biomarker-guided reperfusion therapy for stroke patients. Study results could be used as a basis for economic evaluations of other test biomarkers for IS and/or ICH.

This study has some limitations which need to be considered when interpreting the results. First, the mRS score at month 3 for patients who underwent the biomarker test had to be estimated from another study [15] as results from a clinical trial using biomarkers to guide tPA are not available. However, when theoretically performing the test in the ambulance and applying tPA for patients who meet thrombolysis criteria, the reduction in the delay to thrombolysis is likely similar to that obtained in the Kunz et al. study [15].

Although the assumptions about long-term health results and costs were validated by clinical experts and made in accordance with previous studies, long-term clinical studies in Spain on stroke with reliable clinical data are lacking.

The present study was conducted from the perspective of the Spanish Health System. Nevertheless, it would have been ideal to assess the relative efficiency of the test biomarker from a societal perspective, since informal care plays an important role in stroke, which can reach 70% [24, 45]. Moreover, it was observed that 15% of the patients required home modifications [45]. However, there are no available data on costs incurred by stroke patients in Spain.

In our model, we did not include stroke mimics, which represent a variable proportion (9% to 27%) of patients initially diagnosed with IS [46]. Stroke mimics could decrease test effectiveness, although the test might also help to avoid administering tPA to stroke mimics, which happens occasionally in stroke units. In the Bustamante study [19], when they included stroke mimics, biomarker specificities were reduced to 98.4 and 96.8%. However, stroke thrombolysis in the 3-h window remains cost-effective from the healthcare sector perspective when stroke mimics proportions among arriving patients are less than 30% [47]. In addition, our study is limited to patients with suspected moderate to severe stroke within 4.5 h of symptom onset. Therefore, these results would not be applicable to the whole stroke population and, especially, are not applicable to tPA treatment in extended time windows.

Perhaps, the main limitation of the present study is the fact that clinical results supporting the biomarker data came from a single study [19], which would require external validation. Currently, the BIOFAST study is replicating the biomarker results in a real-world scenario, with blood samples collected at the ambulance and measuring the biomarker panel with a POCT device. If replicated, the BIOFAST study would be supported not just from robust clinical data, but also by the present cost-efficacy study, representing a crucial step for an earlier implementation in healthcare systems.

It is important to highlight the clinical impact of initiating treatment soon after symptom onset in stroke patients. Accordingly, the biomarker test could play an important role in the pre-hospital diagnosis of these patients. It could be relevant to include stroke mimics in the economic model.

## Conclusions

The use of a biomarker test to guide pre-hospital tPA is cost-effective compared with standard care in patients with IS from the Spanish Health System perspective.

## Supplementary Information

Below is the link to the electronic supplementary material.

Supplementary file1 (DOCX 97 KB)
