# Supplementary material for: Modeling the potential efficiency of a blood biomarker-based tool to guide pre-hospital thrombolytic therapy in stroke patients
Source: Eur J Health Econ. 2022 Jul 27;24(4):621–32. doi: 10.1007/s10198-022-01495-1 (PMC10175459; doi:10.1007/s10198-022-01495-1)
Supplement: Supplementary file 1 — Supplementary file1 (DOCX 97 KB) [file 10198_2022_1495_MOESM1_ESM.docx]

**Supplementary material**

**Table 1S.** Diagnostic validity of the biomarker test

| **Panel two biomarkers** |  | |
| --- | --- | --- |
|  | **Ischemic stroke** | **Intracerebral haemorrhagic** |
| **Test +** | 31 | 0 |
| **Test -** | 123 | 34 |

Source: Bustamante et al.^19^

**Table 2S**. Maximum test cost to be cost-effective.

| **Specificity 100%** | Cost (euros) |
| --- | --- |
| Sensitivity 99% | 13986 |
| Sensitivity 60% | 8747 |
| Sensitivity 50% | 7329 |
| Sensitivity 40% | 5946 |
| Sensitivity 30% | 4425 |
| Sensitivity 20% | 3005 |
| Sensitivity 10% | 1688 |
| **Specificity 97%** |  |
| Sensitivity 99% | 10197 |
| Sensitivity 60% | 6187 |
| Sensitivity 50% | 5027 |
| Sensitivity 40% | 4043 |
| Sensitivity30% | 2880 |
| Sensitivity 20% | 1773 |
| Sensitivity 10% | 655 |
| **Specificity 94%** |  |
| Sensitivity 99% | 7850 |
| Sensitivity 60% | 4438 |
| Sensitivity 50% | 3616 |
| Sensitivity 40% | 2745 |
| Sensitivity 30% | 1809 |
| Sensitivity 20% | 874 |
| Sensitivity 10%* | NA |

NA: No apply

*Even when the cost of the test is zero, it is not a cost-effective alternative with these sensitivity and specificity values.

**Figure 1S.** Incremental cost-effectiveness ratio (ICER) according to the sensitivity of the biomarker test with a specificity of 100%

**Figure 2S**. Incremental QALY according to the sensitivity of the biomarker test with a specificity of 100%.

**Figure 3S**. The incremental cost-effectiveness plane
